# Safety Considerations in 3D Bioprinting Using Mesenchymal Stromal Cells

**DOI:** 10.3389/fbioe.2020.00924

**Published:** 2020-10-08

**Authors:** Lucy Belk, Nazzar Tellisi, Hamish Macdonald, Ahmet Erdem, Nureddin Ashammakhi, Ippokratis Pountos

**Affiliations:** ^1^Academic Department of Trauma and Orthopaedics, University of Leeds, Leeds, United Kingdom; ^2^School of Medicine, University of Leeds, Leeds, United Kingdom; ^3^Chapel Allerton Hospital, Leeds Teaching Hospitals, Leeds, United Kingdom; ^4^Gloucester Royal Hospital, Gloucestershire Hospitals NHS Foundation Trust, Gloucester, United Kingdom; ^5^Center for Minimally Invasive Therapeutics, University of California, Los Angeles, Los Angeles, CA, United States; ^6^Department of Bioengineering, Henry Samueli School of Engineering, University of California, Los Angeles, Los Angeles, CA, United States; ^7^Department of Chemistry, Kocaeli University, Kocaeli, Turkey; ^8^Department of Biomedical Engineering, Kocaeli University, Kocaeli, Turkey; ^9^Department of Radiological Sciences, David Geffen School of Medicine, University of California, Los Angeles, Los Angeles, CA, United States; ^10^Institute for Quantitative Health Science and Engineering, Michigan State University, East Lansing, MI, United States; ^11^Department of Biomedical Engineering, Michigan State University, East Lansing, MI, United States

**Keywords:** mesenchymal stromal cells, *ex vivo* expansion, bioprinting, additive manufacturing, 3D bioprinting

## Abstract

Three-dimensional (3D) bioprinting has demonstrated great potential for the fabrication of biomimetic human tissues and complex graft materials. This technology utilizes bioinks composed of cellular elements placed within a biomaterial. Mesenchymal stromal cells (MSCs) are an attractive option for cell selection in 3D bioprinting. MSCs can be isolated from a variety of tissues, can pose vast proliferative capacity and can differentiate to multiple committed cell types. Despite their promising properties, the use of MSCs has been associated with several drawbacks. These concerns are related to the *ex vivo* manipulation throughout the process of 3D bioprinting. The herein manuscript aims to present the current evidence surrounding these events and propose ways to minimize the risks to the patients following widespread expansion of 3D bioprinting in the medical field.

## Introduction

With an increasing aging population the need to regenerate diseased tissues or replace tissues and organs lost due to trauma or surgery is increasing (Colwill et al., 2008; International Population Reports, 2016). There is already a lack of supply of sufficient organ donations and tissue grafts which is likely to worsen in the future (Yanagi et al., 2017; American Transplant Foundation, 2018). Tissue engineering that was introduced in the last few decades generally employs the seeding of scaffolds with cells (Langer and Vacanti, 1993). This process is associated with inhomogeneous distribution of cells within the scaffold, which can also affect subsequent engineered construct survival, integration and function (Gao et al., 2014). It was previously hypothesized that inhomogeneous seeding could prevent some cells from nutrients and oxygen resulting in poor function (Melchels et al., 2010).

**FIGURE 1:**
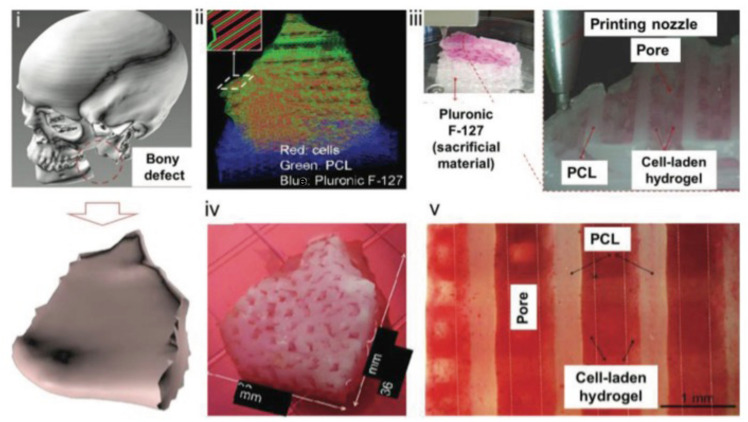
The pathway of creating complex 3D printed structures. **(i)** Modeling of a mandibular defect with the use of patients CT scans. **(ii)** Construction of 3D architecture. **(iii)** 3D printing process. **(iv)** Culture of the graft. **(v)** Differentiation of the cells to osteoblasts. Reproduced with permission from Kang et al. (2016).

The recent advent of three-dimensional (3D) bioprinting has brought about new possibilities to advance tissue engineering and regenerative medicine. Three-dimensional bioprinting involves the use of cells that are mixed with a carrier material while in liquid form with subsequent solidification of such material by using one of a number of cross-linking techniques. This mixture, known as bioink may also include growth factors (Ashammakhi et al., 2019a, b) or other additives such as osteoconductive materials (Byambaa et al., 2017; Ashammakhi et al., 2019c). Three-dimensional bioprinting techniques and bioinks have evolved tremendously over the last two decades, to address the need to create complex biomimetic tissue constructs (Mandrycky et al., 2016; Figure 1).

Cells used in bioinks have represented one of the major challenges faced by tissue engineers because of their limited availability (Freimark et al., 2010), proliferation (Willerth and Sakiyama-Elbert, 2008), and differentiation potential (Tuszynski et al., 2014). While already differentiated cells could be ideal, their harvest can cause donor site morbidity while often perform poorly with ex vivo manipulation. Alternative cell sources of cells include embryonic or reprogrammed cells. These cell types are associated with many challenges (Bongso et al., 2008; Trounson and McDonald, 2015) and concerns. The biggest concern shared by physicians and other care providers, regulatory bodies and industry as a whole is the safety of stem cell therapeutics for use in patients (Goldring et al., 2011). Mesenchymal stem cells on the other hand, have gained popularity and represent a cell type of choice for many experimental and clinical studies in tissue engineering.

## MSCs in 3D Bioprinting

Mesenchymal stromal cells (MSCs) represent one of the most popular types of cells used in tissue engineering today. In fact, their clinical use is so strong today that are used in more than 700 clinical trials listed on US clinical trials. This is because MSCs have potential to differentiate into a wide variety of cell types (Sasaki et al., 2008) but also due of their wide availability from different sources such as the bone marrow (Gnecchi and Melo, 2009), adipose tissue (Katz et al., 2005), blood vessels (Kuznetsov et al., 2001), muscle (Young et al., 1995) as well as rather “embryonic” tissues such as amniotic fluid (Tsai et al., 2004) and cord blood (Bieback et al., 2004). MSCs actively participate in the regeneration of tissues and provide substitute cells for those that expire (Pintus et al., 2018). Following injury MSCs mobilize to distant sites and either provide reparative cells and/or secrete trophic factors to promote healing. In addition, MSCs pose anti-inflammatory and immunomodulatory capacity as can improve inflammation and restore or inhibit the functions of immune cells (Pintus et al., 2018). MSCs can be easily expanded *ex vivo* to provide clinically relevant numbers prior to use. Although their exact function is not fully elucidated, MSCs have been used widely in tissue engineering instead of pluripotent stem cells (embryonic or induced pluripotent stem cells) which possess their own concerns and more complex processing techniques (Porada et al., 2006).

In 3D bioprinting, MSCs remains a popular cell type for the use in bioink. Their use is not limited to bone (Ong et al., 2018), cartilage (Bae et al., 2018), and adipose tissue (Qi et al., 2018) but MSCs are considered and used in many other 3D bioprinting applications. In fact, in addition to bone and cartilage, MSCs were used in 3D bioprinting of muscle (Phillippi et al., 2008), aortic valve (Kang et al., 2017), cardiovascular tissue (Ryu et al., 2015), neural tissues (Jakab et al., 2010), tendons and ligaments (Rak Kwon et al., 2020), and others (Tasnim et al., 2018). Thus, the objective of this review is to examine the literature on 3D bioprinting that utilized MSCs and examine accumulated data pertaining to the safety of MSCs in 3D bioprinting in various pre-, intra-, and post-printing stages. Discussion of findings is included, challenges highlighted, and future directions are outlined.

## Pre-Printing

The generation of reliable MSC-based 3D bioprinting products requires first an in-depth understanding of the MSC physiology. MSC physiology is complex and it is influenced by the local microenvironment. For example, some researchers have shown that MSCs have tumor-suppressing properties (Khakoo et al., 2006; Cousin et al., 2009; Ho et al., 2013). On the contrary, MSCs can also favor tumor progression by promoting tumor angiogenesis, maturation of tumor vasculature and expansion through the secretion of a wide range of bioactive biomolecules (Kucerova et al., 2010; Suzuki et al., 2011; Huang et al., 2013). The reason for such dual roles is largely obscure. Together with MSC physiology, the target tissue micro-architectural topography, physiology, mechanical properties have to be elucidated. This will dictate the porosity, stiffness, orientation of the scaffold components and depict the exact location of the cellular components (Daly et al., 2017).

In addition to robust understanding of MSC physiology, further work on developing methodologies that safeguard high viability and ensure safety of grafts is needed. Literature suggests that the success of potential application of MSCs is closely related to the number of MSCs (Hernigou et al., 2005a, b). The expansion of the cells raises several concerns involving the extent of the expansion (expansion induces deprivation of MSCs properties), the effect of culture conditions, culture media and tissue culture plastics on the cells as well as the effect of cryopreservation on MSCs (Sotiropoulou et al., 2006; Pountos et al., 2007). The need for supplementation of the culture media with cytokines and chemokines in high non-physiologic concentrations is unknown whether it can affect their long-term properties. Worrying reports are available suggesting, that *ex vivo* expansion of MSCs can induce spontaneous malignant transformation into cells with tumorigenic potential (Rubio et al., 2005). Even more disturbing are the reports of occasional sarcoma formation in patients receiving bone marrow treatment and those undergone autologous fat graft (Perrot et al., 2010).

## Printing Process

### Characteristics of 3D Bioprinting Methods in Brief

**FIGURE 2:**
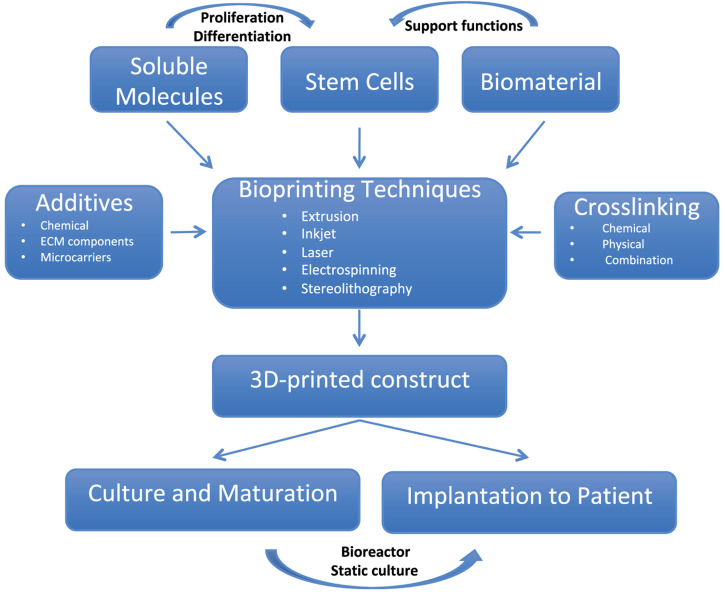
The pathways and components of 3D bioprinting process.

**FIGURE 3:**
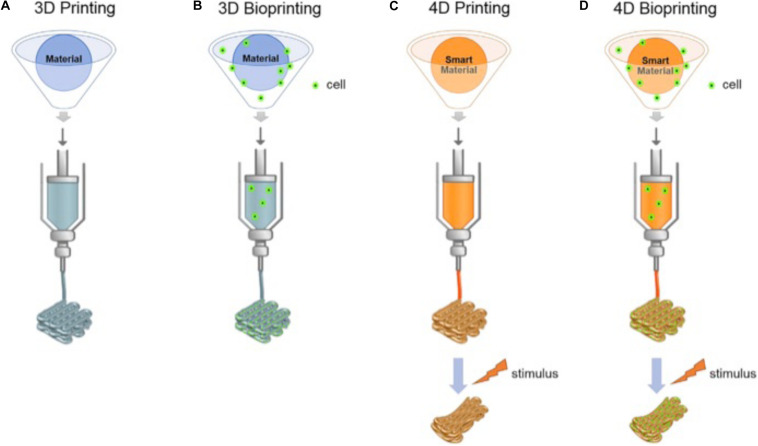
The different printing technologies (3D, 3D bioprinting, 4D, and 4D bioprinting). **(A,B)** shows conventional 3D printing and bioprinting techniques. **(C,D)** For-dimensional bioprinting is defined as 3D printing of cell-laden materials in which the printed structures would be able to respond to external stimulus due to stimuli-responsive bioinks or internal cell forces. Reproduced with permission from Ashammakhi et al. (2018).

There are several 3D printing techniques among which the most commonly used for 3D bioprinting are extrusion, laser-based (Catros et al., 2011), inkjet (Cui et al., 2010), stereolithography (Wang et al., 2015) and electrospinning-based printing (Khalil and Sun, 2009; Wüst et al., 2011; Dababneh and Ozbolat, 2014; Figure 2). The same technologies can be used to create smart 3D-bioprinted structures able to respond to the environment; commonly referred to as four-dimensional (4D) bioprinting (Figure 3). Extrusion 3D bioprinting or pressure-assisted bioprinting uses hydrogel bioinks extruded from a syringe in a continuous trace through a fine nozzle (Maher et al., 2009; Bhuthalingam et al., 2015; Irvine et al., 2015). In most extrusion bioprinters, the nozzle can move on y-z axes with the substrate collector plate moving in the *x*-axis to produce the final structure (Maher et al., 2009; Bhuthalingam et al., 2015; Irvine et al., 2015). Extrusion bioprinting delivers good homogeneity of bioinks, can deliver very high cell densities and does not require any specific environmental conditions (can be carried out at room temperature) (Atala and Yoo, 2015; Bishop et al., 2017). The overall resolution is rather poor compared to other techniques (minimum feature size is generally over 100 μm) (Leberfinger et al., 2017). Despite this, the technique has been used to create complex structures but MSCs survival was as low as 40% due to apoptosis and cell deformation.

Laser bioprinting uses a pulsed nanosecond or ultraviolet (UV) like wavelength laser as a source of energy to stimulate the upper surface of an energy absorbing metal film, which is usually made of a layer of titanium or gold (Catros et al., 2011). This metal film is coated with bioink on its lower surface and acts as a donor film. Stimulation of the upper surface of the metal film causes vaporization, creating a pressure bubble that drives the bioink from the donor film onto a substrate plate containing a biopolymer (Stolberg and McCloskey, 2009; Jana and Lerman, 2015; Irvine and Venkatraman, 2016; Li et al., 2016). The biopolymer functions to aid in sustaining growth and cellular adhesion of the cells after transfer from the donor film (Catros et al., 2011; Trombetta et al., 2017). The precise resolution is influenced by a number of factors including the energy emitted by the laser, printing speed, viscosity and thickness of the bioink layer on the donor film and its rheological properties, shape and organization of the structure and substrate wettability (Guillemot et al., 2010a, b; Li et al., 2016). Despite that, this is a scaffold-free technique reaching resolutions between 10 and 50 μm. Some studies managed to achieve a resolution of a single cell per droplet. This method negates the shearing stress experienced by cells during deposition down a narrow print head or nozzle (Murphy and Atala, 2014; Mandrycky et al., 2016; Keriquel et al., 2017). The potential of laser bioprinting has been demonstrated in a number of studies (Barron et al., 2004; Guillemot et al., 2010a, b).

Inkjet bioprinting arose from the adaptation of conventional desktop inkjet printers. It is a noncontact printing process where a droplet of bioink is deposited through the print head on demand, under the control of a thermal or piezoelectric actuator. This type of multi-cell printing is known as drop on demand (Irvine and Venkatraman, 2016). The resolution is in the region of up to 50 μm (Mandrycky et al., 2016). Thermal actuation is the more commonly used method for inkjet bioprinting where droplets of bioink are generated by an electric current. The thermal actuator element reaches temperatures in excess of up to 300°C, allowing a vapor bubble to generate sufficient pulse pressure to expel the bioink from the print head (Cui et al., 2010). This could potentially impart both shear and thermal stress on the cells (Irvine and Venkatraman, 2016; Li et al., 2016). The requirement to use low viscosity inks to prevent blockage of the print-head prevents the use of a number of efficacious bioinks. In contrast to the thermal, the piezoelectric actuation produces a transient pressure to eject the droplets on to the substrate. It produces more homogenous droplets than thermal actuation, but some authors reported greater levels of cell damage (Seetharam, 1991; Nakamura et al., 2005; Saunders et al., 2007).

Stereolithography is another 3D bioprinting technique that can be used to generate 3D constructs. This technique involves the solidification of a cell-laden photo-crosslinkable polymer solution in a layer-by-layer fashion, and it is controlled by a moveable stage along the *z*-axis (Murphy and Atala, 2014). In stereolithography, 3D complex structures can be produced without the need for a printhead that moves in x–y direction. In this process, a digital micromirror device (DMD) which allows highly precise patterns to be created, is used to control selectively crosslinking of bioink in z direction (Heinrich et al., 2019). This selective crosslinking method by light does not lead to any cell shear stress, making it possible to achieve higher cell viability in produced constructs. However, the use of transparent bioinks is required in stereolithography in order to achieve uniform crosslinking. This restricts the cell density that can be used in the bioink (Minteer et al., 2013). Despite this limitation, the technique has a great potential because of high speed, high resolution (∼1 μm) and controllability of the internal and external architecture of the resulting construct (Gruene et al., 2011a, b; Kang et al., 2017).

Electrospinning is a high-resolution fabrication method that can be used to produce thin fibers (Heinrich et al., 2019). During the process of electrospinning, a high voltage is applied to the ejected polymeric solution from the syringe. When the electrostatic repulsion starts to overcome the surface tension of the solution, the solution begins to evaporate and it is subsequently solidified during transit to form fibers (Ashammakhi et al., 2008; Bhardwaj and Kundu, 2010). Thin fiber-based constructs can be produced by this technique. Recently, this process technique has been modified for bioprinting by adding cells and controlling the process of fiber arrangement in the resulting structure. One of the primary features of electrospinning-based bioprinting (EBB) is the shorter collecting range of fibers (around 0.5–3 mm) in comparison to traditional electrospinning. This allows for more controllable deposition of electrospun materials with less applied voltage than usually used in conventional electrospinning (Heinrich et al., 2019). Visser et al., 2015; have recently used electrospinning-based bioprinting technology to enhance GelMA hydrogel mechanical strength by reinforcing high-porosity poly(ε-caprolactone) (Visser et al., 2015). The rigidity of GelMA hydrogels increased 30 times by 7–214 kPa while its elastic properties were preserved (Visser et al., 2015). However, the main restrictions of EBB are the fast spinning of fibers, resulting in a spatially unstable 3D structures and the high processing temperature and voltage, which is challenging to cells contained in the electrospun material.

### Cell Death During 3D Bioprinting

The viability of the cells can be influenced by a number of factors. These include the storage of the cells in the printer, the thermal damage during the printing process and the mechanical forces exerted during bioprinting. Table 1 shows documented survival rates following 3D bioprinting.

**TABLE 1:** Studies presenting the survival rates of cells used as bioink for 3D-bioprinting applications.

Author, year	3D Printer	Cell types	Survival rates	Comments/Other findings
**Inkjet bioprinting**				
Christensen et al. (2015)	Thermal inkjet printing	Chinese hamster ovary cells and primary embryonic motor neurons from ventral cords of 14-day embryos from pregnant Sprague-Dawley rats	Greater than 90% cellular viability after printing.	
Saunders et al. (2007)	Piezoelectric drop-on-demand inkjet printing	HT 1080 human fibroblasts	Cellular survival of 94–98%.	Survival rates decrease with increased printing pulse amplitude. Sampled printed at 40v demonstrated survival rates that could not be distinguished from unprinted control samples.
Cui et al. (2010)	Thermal inkjet printing	Green fluorescent protein expressing Chinese hamster ovary cells	Average cellular viability was 89%.	No significant difference in viability was observed in different cellular concentrations of ink. Printed cell number correlated with increasing cellular ink concentrations.
Christensen et al. (2015)	Inkjet based free form fabrication	NIH 3T3 mouse fibroblasts	Post printed cellular viability was 92.4% immediately after printing and 90.8% after 24 h of incubation.	
Levato et al. (2014)	Bioscaffolder system (Levato et al., 2014)	Mesenchymal stem cells from 2 to 4 weeks old Lewis rats	Post dispensing viability was 80% after 1 day and more than 90% after 3 days.	Pre-seeded particles suspended in the gels had the lowest number of viable cells (60%) after 1 day of culture, which increased to 90% after 3 days.
Du et al. (2015)	Inkjet with four independent *z*-axis-controlled ink reservoirs	Bone mesenchymal stem cells from 4-weeks-old male adult Sprague-Dawley rats	Cellular viability of > 90% was seen during printing	CBD-BMP2-collagen microfibers induced BMSC differentiation into osteocytes within 14 days more efficiently than the osteogenic medium.
**Extrusion bioprinting**				
Zhao et al. (2014)	Microextrusion printing	HeLa cells	Post printed viability of the HeLa cells in constructs was 94.9% ± 2.2% with parameters of 10 mm^3^ min^–1^ extrusion speed, 250 μm nozzle inner diameter, 10°C chamber temperature and 25°C nozzle temperature.	Comparisons of 3D and 2D tumor models of HeLa cells show a higher cellular proliferation rate and more simulated tumor characteristics with 3D printing
Zhao et al., 2015	Four nozzle microextrusion printing	A549 cells	Cell survival rate was > 90% for all rheological conditions at a holding temperature of 20°	For all concentrations of bioink used in microextrusion printing, a holding temperature of 20° should be used. Optimum holding times were variable, dependent upon bioink concentration
**Laser assisted bioprinting**				
Barron et al. (2005)	BioLP^TM^ Biological Laser Printing	Human osteosarcoma cells	After six days of incubation, cells demonstrated a 100% viability	
Koch et al. (2010)	Laser based printing based on laser assisted forward transfer (LIFT)	Skin cell lines (fibroblasts, keratinocytes); Human mesenchymal stem cells	98% ± 1% standard error of the mean (skin cells) and 90% ± 10% (hMSC).	No increase in apoptosis or DNA fragmentation was seen with the use of LIFT. hMSC phenotype was maintained as proven by fluorescence activated cell sorting analysis.
Hopp et al. (2012)	Femtosecond KrF laser in laser assisted forward transfer (LIFT)	Human neuroblastoma, chronic myeloid leukemia and osteogenic sarcoma cell lines and primary astroglial rat cells	Short-term and long-term survival for neuroblastoma and astroglial cells was 65–70%. Long term survival of osteosarcoma cells was low, while myeloid leukemia cells did not tolerate the procedure under the conditions.	
**Stereolithography bioprinting**				
Arcaute et al. (2006)	Stereolithography bioprinting	Human dermal fibroblasts	Cell viability was at least 87% at 2 and 24 h following fabrication.	
Raman et al. (2016)	High-resolution projection stereolithography bioprinting	fibroblasts (3T3), myoblasts (C2C12), endothelial (C166), and bone marrow stromal (D1) cells	Cells encapsulated in the lower molecular weight polymer demonstrate a viability of 70% ± 10%, whereas cells encapsulated in the higher molecular weight polymer demonstrate a viability of 93% ± 3% on day 1 after printing for 3T3 cells. In the long term (2 weeks) cell viability in low molecular weight does not significantly change, but cell viability in high molecular weight significantly increases.	
**Electrospinning-based bioprinting**				
Visser et al. (2015)	Electrospinning-based bioprinting	Chondrocytes	Chondrocytes maintained high cell viability (∼80%) on days 1 and 7.	

Cell storage and conditions during the printing process can potentially affect cell viability. During this process the cells are required to be stable and in media that could allow them to recover from the effects of cells-detaching solution (i.e., Trypsin, TrypLE, collagenase or others) and the stress exerted on them during the detachment process (i.e., centrifugation, washing, etc.). It is known that these methods can affect cell survival, phenotype and differentiation potential (Parvin et al., 2012; Tsuji et al., 2017). In addition, the effect of prolonged bioprinting protocols would require stable media and stable cell conditions. At present, there are limited studies in this field.

Thermal injury to cells is another area of concern. For example, during inkjet printing, where temperatures exceed 200 °, studies have shown that the bioink temperatures are raised by just 4–10° (Cui et al., 2010) and this does not significantly adversely affect the viability of mammalian cells (Suzuki et al., 2011). This heating effect is thought to be temporary (∼5 μs), with less than 8% of the cells being lysed during bioprinting (Cui et al., 2010). Similar results were reported for the heat shock of the laser pulse where the cell survival, proliferation and differentiation were comparable to those of controls at 5 days in cell culture (Gruene et al., 2011b).

In addition to the potential thermal damage, the mechanical stress should be also taken into account. Cells are known to respond to mechanical stress by changing their gene expression and cell function. Among many cells’ adaptation mechanisms activated, MSCs activates several intracellular signaling cascades, including kinases (PKB, MAPK, FAK), *β*-catenin, GTPases (Thompson et al., 2012). Chang et al. (2008), found that cellular viability is inversely related to extrusion pressure, with as little as 40% viability found at the extremes of high pressure. Mechanical pressure observed in inkjet printing has been demonstrated to promote the differentiation of MSCs toward bone and cartilage lineages (Shav and Einav, 2010). In contrast, the shear stress produced in extrusion techniques promotes differentiation toward both endothelial and bone tissues (Stolberg and McCloskey, 2009). The choice of the 3D technology is mostly done on the basis of required resolution and the target tissue as well as other factors. Lee et al. (2015) suggested that laser assisted and inkjet bioprinting may be preferable to extrusion bioprinting in most circumstances, but where circumstances necessitate the use of bioink with a high viscosity, extrusion bioprinting may be necessary. In these circumstances, the effects of sheer stress may be countered by modification of the bioink composition, e.g., by the inclusion of thinning polymers and the control of back pressure during the printing process (Mackay et al., 1998).

### Bioink Characteristics and Cellular Adhesion

The primary aim in preparing a bioink is the biomimicry of the extracellular matrix, which creates a microenvironment that is optimal for cellular adhesion, proliferation and differentiation. An ideal bioink will maintain its printed structure integrity, be crosslinkable and can undergo degradation. It must accommodate cells, and sustain their integrity and viability throughout the printing process (Irvine and Venkatraman, 2016; Grungor-Ozkerim et al., 2018). It should also have the specific mechanical, physicochemical, rheological and biological properties needed for printability and for the preservation of cellular phenotype (Byambaa et al., 2017). Skardal and Atala (2015) highlighted that most biocompatible bioinks which were able to bear the vertical weight of emerging structures either produced toxic macromolecules during the setting process or required a toxic solvent for setting itself.

Porosity and interconnectivity are also two essential factors. Pore size, shape and volume are all influential in the behavior of cells following adhesion to the scaffold structure. Matsiko et al. (2015), found that pore size correlates with cellular organization, mineralization and the development and assembly of collagen I. Greater porosity and more interconnectivity allow for better matrix deposition and transportation of oxygen and other essential substrates into the center of the scaffold, promoting better ingrowth of tissue. Domingos et al. (2013), concluded that the morphology of printed cells did not appear to be influenced by the topology of pores, but that cell viability and proliferation were strongly affected by the size and shape of the pores, with large quadrangular pores resulting in the best viability and proliferation of human MSCs.

Scaffold stiffness has also been noted to play an integral role in the terminal differentiation of cells. MSCs have been observed to differentiate into cell types that best fit the microenvironment supported by the mechanical properties of the attachment surface or matrix. Differentiation toward an osteogenic lineage is observed in cells adhering to a rigid surface (34 kPa), compared with a more elastic surface (0.1–1 kPa), where MSCs display a tendency to differentiate toward a neuronal lineage (Engler et al., 2006; Lane et al., 2014). In relatively soft hydrogels (2.5–5 kPa), a differentiation toward adipogenesis is observed (Arany et al., 2010). This offers the possibility for the modification of bioink matrices and scaffolds to induce a specific lineage differentiation. Gao et al. (2015), produced a bioink that was optimized for bone and cartilage regeneration. The ink, made from a hybrid of polyethylene glycol and gel dimethylacrylate, had a compressive modulus of 1–2 MPa when printed, significantly stiffer than previously used hydrogels. MSCs printed in this hydrogel demonstrated a greater propensity toward osteocyte and chondrocyte lineage (Gao et al., 2015), but only in the context of specific extracellular matrix (Rowlands et al., 2008) and cross-linking conditions (Das et al., 2015).

It has been previously suggested that a scaffold can guide MSCs toward a specific lineage. In cases where the aim is to maintain stemness, bioinert hydrogels should be used. This avoids creating an environment that may be favorable to one particular lineage of cells. One such example of a bioinert hydrogel is alginate (Irvine and Venkatraman, 2016) which retains the stemness of printed stem cells (Blaeser et al., 2016). However, caution must be exercised when using bioinert hydrogels, as proliferative capabilities and movement are reduced, which may promote anoikis (Carrow et al., 2015), however, this may be overcome by the addition of the integrin binding peptide arginyl-glycyl-aspartic acid (RGD) moieties to bioinert alginates which increases cellular interaction whilst maintaining stemness (Carrow et al., 2015). Hyaluronic acid is an alternative to alginate, with proven clinical efficacy (Ozbolat and Hospodiuk, 2015). In contrast to alginate, hyaluronic acid promotes MSC attachment and maintains multipotency and proliferation through CD44 receptors (Cao et al., 2016), with the added benefit of adaptation to promote a specific lineage differentiation. One such example is the use of hyaluronic acid in cardiogenesis (Mairim et al., 2012). Where bioinert inks have been used, MSCs can be differentiated by incubation with soluble factors that direct maturation to a specific lineage in a similar fashion to culture additives (Irvine and Venkatraman, 2016). To remove reliance on extrinsic factors, additives can be included in bioink. For example, alginate bioinks have been modified with the addition of hydroxyapatite in the context of bone regeneration (Wüst et al., 2014). *In vivo* murine models of alginate scaffolds containing biphasic calcium phosphate particles (consisting of hydroxyapatite and β-tricalcium phosphate) displayed greater osteogenic differentiation than scaffolds having no biphasic calcium triphosphate (Wang et al., 2007).

### The Effect of Cross-Linking

Three-dimensionally bioprinted extracellular matrix may lack the required stability and integrity to support contained cells. Crosslinking is often an essential step and a number of physical, biological and chemical crosslinking techniques have been proposed over the years. The aim of these techniques is to enhance the mechanical and biological properties of the grafts preventing the cell-mediated contraction. Crosslinking induces chemical or physical links between the polymer chains of the scaffold and can be achieved by using UV light, dehydrothermal treatment, or treatment with sodium citrate, sodium tripolyphosphate, sulfosuccinic acid, oxalic acid, glutaraldehyde, genipin, or carbodiimide (Lew et al., 2007; Pfeiffer et al., 2008; Jóźwiak et al., 2017; Vining et al., 2019).

Crosslinking can affect several of the cellular functions, including proliferation, differentiation and cellular ability to attach to a scaffold (Davidenko et al., 2015). Kim et al., investigated the effect of different crosslinking techniques on immortalized human corneal epithelial cells, human skin fibroblasts, primary bovine corneal endothelial cells and immortalized human retinal pigment epithelial cells (Kim et al., 2014). The authors reported different toxicity levels with the least toxic being with mononitroalcohols and glyceraldehide, intermediate toxicity being with nitrodiol and nitrotriol, and highest toxicity being with glutaraldehyde, paraformaldehyde, genipin, and bronopol. Several studies have also defined the critical concentration over which the agent induces cytotoxic effect (Wang and Stegemann, 2011; Muzzarelli et al., 2015). On the contrary, some studies suggest that crosslinking can have a positive effect on cellular function. Raucci et al. (2015), studies the effect of citric acid crosslinked cellulose containing hydrogel on the osteogenic differentiation of MSCs. The authors revealed enhanced hydrophilicity and roughness of the hydrogel together with a stimulation of osteogenic differentiation as demonstrated by enhanced expression of bone markers such as osteopontin and osteocalcin. In addition to the direct effect of the crosslinking on MSCs, the physical properties of the extracellular matrix can regulate the response and phenotypes of the cells (Kyle et al., 2019).

Despite many promising studies, to date, there is no gold standard method for cross-linking 3D printed biomimetic materials. In cases where multiple bioinks are used, tuning the scaffold microstructure through crosslinking of multiple biomaterials without affecting its properties will require significant improvement in our 3D printing technology. In tissues where biodegradation or regeneration is required, like for example in 3D bioprinting of bone, the mechanical properties of scaffolds are negatively correlated with their biodegradation profile (Oryan et al., 2018). Finally, one major concern is the potential inflammatory reaction following implantation. It is shown that the cross-linking methods can induce an immune reaction, initiate M1 macrophage response and inhibition of M2 macrophage polarization, reduced cell infiltration, increased proinflammatory cytokine expression and peri-implantation fibrosis (Delgado et al., 2015), which should be carefully considered and solutions devised.

## Post-Printing

Following 3D printing, cell-laden scaffolds will require incubation prior to implantation. This raises the question of how the nutrients and wastes will be exchanged to support the cells until implantation. For a thin construct, this can be done through a static culture through diffusion; however, functioning vasculature will be required for larger constructs. Dynamic culturing can provide continuous infiltrating flow of medium and/or compressive/tensile loading, which is most beneficial for cartilage and bone tissue engineering (Butler et al., 2009). In case the technology reaches the stage of creating vasculature (Shahabipour et al., 2020), research would be needed to determine if blood would be an adequate medium to facilitate nutrients and waste exchange.

In addition to the nutrient supply, cells will require time to attach onto the scaffold. It has been previously shown that post-fabrication incubation for long periods can increase the mechanical strength of the construct due the function of the cells and further tissue development (Butler et al., 2009). If photopolymerization is used to harden the bioink, it is unknown whether it can cause cytotoxicity due to the photoinitiators and ultraviolet light. Visible light-sensitive photoinitiators are reported to cause less cytotoxicity but this area is poorly explored (Lim et al., 2016; Mondschein et al., 2017).

### Future Directions and Conclusion

Three-dimensional bioprinting technology has achieved growing popularity for its favorable potential. There is impressive progress with the pertinent techniques supporting the view that in the near future organ manufacturing will be a reality. Three-dimensional bioprinting can find application in organ and graft transplantation by overcoming the issues of immune rejection and reducing the cost of grafts and could be used to establish platforms for research and drug screening.

MSCs are one of the most popular cell type in tissue engineering and are involved in more than half of the clinical trials since 2000 (Yuan et al., 2019). These cells are most likely to be the main component of 3D bioprinting. In order to preserve and deliver MSCs advantages, it is essential to mimic there *in vivo* microenvironment throughout the 3D biofabrication process (Baker and Chen, 2012). In addition, the availability of nutrients and oxygen remains high and similar to that in the body (Melchels et al., 2010; Ashammakhi et al., 2020). This seems to be the only way for the cells retain their phenotype, adhesion, metabolism, and response signaling (Baker and Chen, 2012).

Despite the great progress we have seen in understanding the biology of target tissues in humans, our knowledge is still based on animal biology. Understanding MSC biology is also crucial and it is in fact the most difficult challenge. This will allow us to direct the efforts creating more physiologically relevant structures. MSCs for example could be used in high densities when creating biomimetic cartilage and bone tissues or in lower densities as supporting cells in other applications. Before, however, we are in a position to discuss such matters we would have to decode our biology in health and disease in humans raises significant ethical issues. Once 3D bioprinting reaches a position of manufacturing complex biomimetic tissues, such as organs and large grafts, an appropriate regulatory framework will be required. Hints that this is imminent are shown in many studies which produced complex grafts. Ethical issues include the ownership of prototypes, the harvesting and type of cells and biomaterials, research as well as commercialization of produced constructs. Regulation in terms of safety is also needed including the biocompatibility of bioinks, long-term safety of grafts and the *ex vivo* manipulation of cells.

The optimal *ex vivo* conditions prior to printing should be established. In our view, minimizing the *ex vivo* journey of the cells is crucial. Harvesting and printing the cells in the same sitting could only be done with knowledge of specific markers for MSC, which we lack at present. This is feasible for other cell types with, such as for example the hematopoietic stem cells, which are currently used without manipulation in cancer patients following whole body irradiation (Bazinet and Popradi, 2019). For MSCs however, at present there is a lack of robust techniques for cell isolation and purification that do not affect MSCs biology and then cell preservation strategies. To this end, one of the major drawbacks is the unavailability of reliable culture media, as current research is merely based on animal derived sera. Serum free media or the use of autologous serum can be an alternative but further research is needed in this matter. In addition, the identification of biomimetic matrices mimicking the native tissue composition and allowing cellular growth and differentiation is required. Finally, conditions under which the 3D constructs will survive following printing potentiate dangers and can jeopardize the whole process. A solution would include developing new bioinks and bioprinters that allow high-resolution fabrication process would diminish the need for post-fabrication culture. Only addressing the aforementioned challenges will safeguard the feasibility and safety of 3D bioprinting for regenerative medicine applications.

## Author Contributions

LB, NT, HM, AE, NA, and IP contributed to the research, writing, editing and formatting of the manuscript. All authors contributed to the article and approved the submitted version.

## Conflict of Interest

The authors declare that the research was conducted in the absence of any commercial or financial relationships that could be construed as a potential conflict of interest.
